# A Bibliometric Analysis of Human-Machine Interaction Methodology for Electric-Powered Wheelchairs Driving from 1998 to 2020

**DOI:** 10.3390/ijerph18147567

**Published:** 2021-07-16

**Authors:** Xiaochen Zhang, Lanxin Hui, Linchao Wei, Fuchuan Song, Fei Hu

**Affiliations:** 1 Department of Industrial Design, Guangdong University of Technology, Guangzhou 510090, China; xzhang@gdut.edu.cn (X.Z.); 2111917097@mail2.gdut.edu.cn (L.H.); 3116002285@mail2.gdut.edu.cn (L.W.); 2 F11X Silicon Photonics Process Development, Intel Corporation, Albuquerque, NM 87124, USA; fuchuan.song@intel.com

**Keywords:** EPW driving, HMI methodology, bibliometric analysis, research status, emerging trends

## Abstract

Electric power wheelchairs (EPWs) enhance the mobility capability of the elderly and the disabled, while the human-machine interaction (HMI) determines how well the human intention will be precisely delivered and how human-machine system cooperation will be efficiently conducted. A bibliometric quantitative analysis of 1154 publications related to this research field, published between 1998 and 2020, was conducted. We identified the development status, contributors, hot topics, and potential future research directions of this field. We believe that the combination of intelligence and humanization of an EPW HMI system based on human-machine collaboration is an emerging trend in EPW HMI methodology research. Particular attention should be paid to evaluating the applicability and benefits of the EPW HMI methodology for the users, as well as how much it contributes to society. This study offers researchers a comprehensive understanding of EPW HMI studies in the past 22 years and latest trends from the evolutionary footprints and forward-thinking insights regarding future research.

## 1. Introduction

According to the World Health Organization (WHO), globally, 75 million people needed a wheelchair by 2018 [1]. Moreover, due to the aging population and the growth of noncommunicable diseases, the need for wheelchairs is increasing. The electric power wheelchair (EPW), as an assistive device for mobility, may enhance the mobility capability and experience of the elderly or the disabled. The EPW human-machine interaction (HMI) methodology, referring to the two-way information exchange of various symbols and actions between the user and the EPW system, plays a key role in EPW design. Although conventional manual control, such as joysticks, dominates the HMI methodology in commercial EPWs, other multimodal HMIs had been persistently studied in research and potentially contend for the leading role over conventional ways.

HMI is an essential technology, especially for the EPW. With the rising need for better life quality for all, there has been an increasing demand for affordability, ease of use, safety, and humanization for the EPW and its HMI. Vigorous development of autonomous ground vehicle and multimodal perception technology has led to an upgrade of the potential of the human-machine system’s capability and accordingly the driving behavior. Moreover, it has gradually transformed, from an assistive equipment to carry people here and there, to a hybrid human-machine collaboration partner with diverse merits from the aspects of extended functionality, usability, feasibility, and social-technology. In most cases, the HMI defines how people cooperate with the EPW; the effectiveness can affect the adoption of the entire system, or even impact the overall success or failure of an EPW. Therefore, it is important to conduct the HMI research for the EPW, since the dedication on HMI implies not only the positive attitude regarding the latest advances in perception and information conveying technology but also the understanding of users and their needs.

Due to the advent of powerful low-cost computer equipment, an expanding application and in-depth research of HMI technology, the development of robotics, and growing recognition of the needs and potential of the disabled, more attention has been focused on the study of EPW HMI methodology, and numerous academic outputs have been published accordingly. These studies apply the latest progress in technology or human-centered design frameworks to conduct direct and in-depth investigations into specific EPW HMI scenarios. Therefore, a systematic analysis that presents an overview of the evolving research over the past few decades and conveys the latest progresses of EPW HMI is essential. However, to the best of our knowledge, there is still a lack of more systematic quantitative review work on the EPW HMI. Thus, this paper concentrates on conducting a visualized bibliometric analysis of academic publications from the Web of Science (WOS) to determine the development status, contributors, hot topics, and latest trends.

The contributions of this work are listed as follows:

(1) To the best of our knowledge, our work is the first up-to-date systematical review work on relevant HMIs for the EPW which is essential in the EPW human-machine loop and socio-technical meaning.
(2) This work interprets the EPW HMI objective quantitative bibliometric analysis, which differs from the conventional qualitative review. It presents and analyzes the essential aspects of the corresponding bibliometrics, including Journal co-citation map, collaboration-ship, co-authorship, co-citation-ship of author and references, and keywords’ co-occurrence-ship.
(3) This work focuses not only on the aspects of engineering and intelligence, but also includes relevant works from the field of socio-technical system design and interaction design. Compared with review works from a purely engineering viewpoint, our work is solid regarding interdisciplinary research and cooperation.

**Figure 1:**
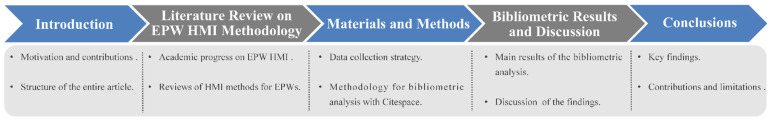
The structural outline of this article.

As shown in Figure 1, the remainder of this paper is organized as follows. In the next section, we provide a literature review about EPW HMI methodology. Then, we describe the data collection procedure and analysis methods. Next, we present the main results of the bibliometric analysis and a discussion of the findings. Finally, we conclude the key findings and explain the contributions and limitations of this work.

## 2. Literature Review on EPW HMI Methodology

Due to the advancement of speech recognition, pupil detection, computer vision, electro-ophthalmogram (EOG), electroencephalogram (EEG), and electromyography (EMG) technologies, there are numerous academic reports on EPW HMI research. Cooper et al. compared the isometric joystick with a conventional position-sensitive joystick during a driving task in a virtual environment and a real environment. The isometric joystick converts the force vector exerted by the operator’s hand into the magnitude and direction of the input, having the possible advantage of reducing the cognitive overhead normally required to monitor joint orientations and torques plus the inertia generated by the mobile limb. Their study found that performance with an isometric joystick and a conventional position-sensing joystick was similar while performing selected driving tasks in both virtual and real environments, supporting additional testing of the isometric joystick as an interface device [2]. Barea et al. introduced an EOG-based eye control method that can be used to guide and control wheelchairs [3]. By combining EOG and EMG technologies, Tsui et al. proposed an EPW hands-free control system that can analyze EMG signals of eyebrow movement and EOG signals of eye movement and convert them into steering control commands (forward, left, right, etc.) for wheelchair driving [4]. Faria et al. used voice commands, facial expressions, head movements, and joysticks as main inputs to control wheelchairs through a flexible multimodal interactive interface. They evaluated the use of the smart wheelchair (SW) in real and simulated environments to demonstrate its practicability and usability [5]. Grewal et al. introduced a new type of automatic wheelchair with a sip-and-puff (SnP) user interface, which can alleviate users’ fatigue compared with traditional SnP-controlled wheelchairs [6]. Kim et al. applied the tongue drive system (TDS) to drive the wheelchair, giving people with severe motor impairment access to a computer and a wheelchair. They proved that the TDS has a better performance in terms of speed and accuracy than the SnP system [7,8]. Iturrate introduced a non-invasive brain-powered wheelchair using a P300 neurophysiological protocol and automated navigation [9]. Long et al. introduced the hybrid brain-computer interface (BCI) to solve the problem of previous BCI systems not providing the multiple independent control signals needed for the continuous control of wheelchairs. The hybrid BCI provides control commands with higher accuracy for users [10]. These studies prove the usability of EPW driving for severely disabled people, improving the mobility experience of users and helping the disabled and the elderly achieve self-independence.

There are also some reviews of HMI methods for EPWs. Lebedev reported that brain-machine interfaces (BMIs) have undergone rapid development in recent years and have a broad range of clinical goals, in addition to enhancing normal brain functions [11]. Phinyomark et al. reviewed the latest EMG-controlled EPW technologies and various EMG-based control methods and summarized the achievements of EMG [12]. Simpson et al. summarized the current technical status of SWs and the direction of future research. Leaman et al. gave a complete overview of the research trends in SWs, including input methods [13,14]. They integrated and analyzed numerous individual studies. Most, however, were expert dependent, and, to a certain degree, this individual preference leads to a lack of objective, systematic quantitative analysis in this field. Thus, this paper concentrates on filling the gap in EPW HMI methodology research by performing a visualized bibliometric analysis of academic publications in this field.

## 3. Materials and Methods

### 3.1. Data Collection

The bibliometric records were retrieved from the WOS, which is considered an ideal data source for bibliometric investigations, with approximately 12,000 worldwide leading journals [15]. We arbitrarily chose the leading core database from the WOS. Specifically, the citation indexes used were the Science Citation Index Expanded (SCI-EXPANDED), the Social Sciences Citation Index (SSCI), the Arts & Humanities Citation Index (AHCI), the Conference Proceedings Citation Index—Science (CPCI-S), and the Conference Proceedings Citation Index—Social Science and Humanities (CPCI-SSH) of the WOS Core Collection. The data retrieval strategy used the following keywords for the period of 1998–2020: TS = (Wheelchair * control OR Wheelchair* driving) AND (Interaction OR Interface). To be specific, the criteria are submitted via regular expressions via advanced filtering, a search engine of Web of Science that allows using field identifiers, Boolean operators, parentheses, and search result sets to create search expressions, while the nonrelated subjects in terms of research area and research practice are occluded. The data were retrieved on 26 October 2020. As a result, 1154 publications on EPW HMI methodology research were collected.

As shown in Table 1, the 1154 publications identified five types of documents, among which proceeding papers (662) are the most frequently used document type, accounting for 57.37% of the total publications. These are followed by articles (451), accounting for 39.08% of the search results. Other document types include reviews (38, 3.29%), letters (2, 0.17%), and meeting abstracts (1, 0.09%). The most cited article is “How Many People Are Able to Control a P300-Based Brain-Computer Interface (BCI)?”. The article mentions the application of EEG-based brain-computer systems in controlling external devices, such as computers, wheelchairs, or virtual environments [16]. The most cited review is “Combining Brain-Computer Interfaces and Assistive Technologies: State-of-the-Art and Challenges”. In this paper, Millan et al. reviewed four application areas where the disabled can greatly benefit from BCI technology: communication and control, motor substitution, entertainment, and motor recovery. Among them, applications in the field of motor substitution include helping users move by directly controlling wheelchairs using the brain or by mentally driving remote mobile robots equipped with obstacle avoidance sensors [17].

The search results were exported with “Full records and Full contents” stored in “txt” format as the input source for CiteSpace.

### 3.2. Analysis Methods

For the objectivity of research results, we used bibliometric methods and CiteSpace software to provide guidance for analysis in the research field through visual information. Bibliometrics is a method of quantitatively analyzing publications using mathematics and statistics. It has been widely used to analyze influential journals, countries, institutions, authors, research hotspots, and frontier trends in many research fields, such as ecological engineering, environmental science, and medicine [18,19,20,21]. Several software packages have been used in the past for bibliometric analysis, each with different capabilities and limitations. Some of the most popular tools include Publish or Perish, CiteSpace, HistCite, and BibExcel [22]. CiteSpace was chosen for this study due to its convenience to identify hidden information through network patterns, such as finding research hotspots and fast-growing areas. Its co-occurrence map of authors, institutions, countries, and keywords and the co-citation map of cited journals, authors, and references can be used to explore the associations between authors, institutions, and countries and analyze contributors, landmarks, hot topics, and research frontiers in a particular research field. The visual map obtained by CiteSpace bibliometric analysis is composed of nodes and links. The links represent the cooperation/co-occurrence or citation relationships between the elements represented by the nodes. The size of the nodes reflects the number of publications or frequency (i.e., citation count). Nodes with a larger centrality, represented by purple on the node ring, are more likely to become key nodes in the network. The different colors of nodes and lines represent different years [23,24,25].

The parameters of CiteSpace were set as follows: time slicing (1998–2020), years per slice (1), term source (all selection), node types (cited journal, country, institution, author, cited author, reference, keyword), selection criteria (threshold C(citation threshold) = (2, 2, 20), CC(co-citation threshold) = (4, 3, 20), CCV(co-citation coefficient threshold) = (4, 3, 20)/(2, 2, 20)), and visualization (cluster view/time zone view).

## 4. Bibliometric Results and Discussion

**Figure 2:**
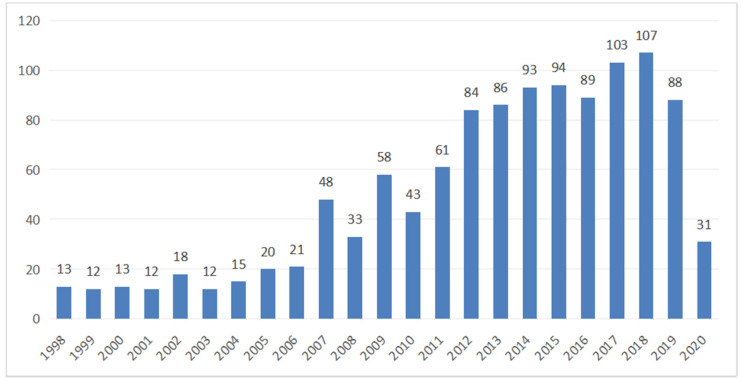
The number of EPW HMI methodology research publications indexed by the Web of Science from 1998 to 2020.

The progression of documents related to EPW HMI methodology, published from 1998 to 2020, is shown in Figure 2. The number of publications has reached a plateau at approximately 95 publications per year in recent years, more than six times the average number of publications from 1998 to 2006. Since the development of HMI-related technology, the advent of powerful low-cost computer equipment, and growing recognition of the needs and potential of the disabled, more studies related to EPW HMI methodology are ongoing.

The number of publications gets doubled from 2007/2008 to 2012/2013, and continuous to grow after. Nowadays, we observe that the low-cost mobile computing terminal and artificial-intelligence-powered autonomous driving technology have gained enormous attention by the field, and as there starts to arise more studies regarding autonomous driving for ubiquitous travel motors, EPW is among the most promising due to its unique properties, such as having a high priority for driving, being pedestrian sidewalk drive-able, and having no license plate required in most countries.

There were two sharp increases in the number of publications after 2006 and 2011 and a sharp dip in 2020. We believe that the first jump may have been due to the development of BCI technology and the prosperity of brain-controlled wheelchair studies, while the second jump was because of neurophysiological protocol applications, algorithm optimization, and control strategy progress. We also suspect that the decline in the publication number in 2020 may be due to publication delays.

### 4.1. Mapping and Analysis by Journal

Knowledge mapping can provide information about professional journals related to a specific research field and thus facilitate researchers to find the key literature. A journal co-citation map with journal co-citation frequency and centrality was used in this study to find influential journals that contributed the most to EPW HMI methodology research.

**Figure 3:**
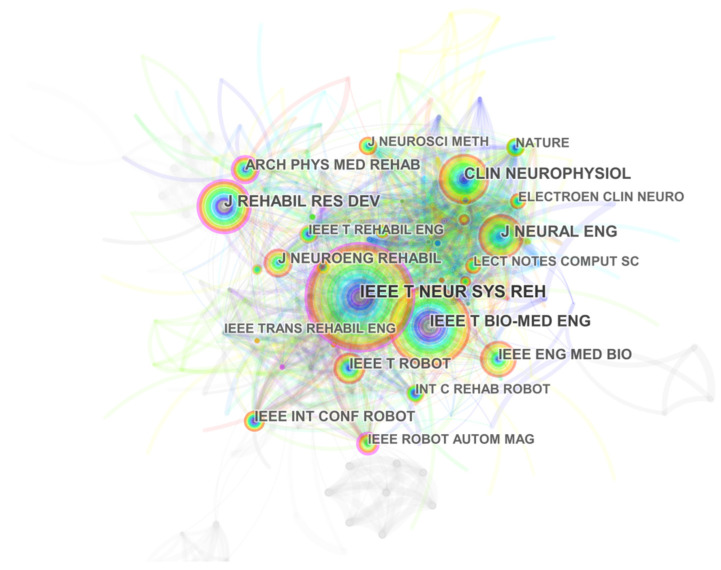
Journal co-citation map related to EPW HMI methodology research from 1998 to 2020.

As shown in Figure 3, 275 different journals from the fields of computer science, biomedical engineering, robotics, rehabilitation and medical engineering, neural engineering, and mathematics were found, indicating that the research and development of EPW HMI methodology is an interdisciplinary problem. In Figure 3, nodes represent the cited situation of journals, the larger the node, the higher frequency of citation of the journal.

Furthermore, five journal sets with co-citation counts of over 248 and five journal sets with a centrality of over 0.10 are listed in Table 2. The journal statistics in Figure 3 and Table 2 reveal the journals that have the most publications and contribute the most to the field: IEEE T Neur Sys Reh is the most prominent, with 469 co-citations, followed by IEEE T Bio-Med Eng (365), Clin Neurophysiol (272), J Rehabil Res Dev (263), and J Neural Eng (248). In terms of centrality, J Rehabil Res Dev has the highest centrality (0.19), and articles in it have been cited since 1999. Other journals with high centrality are IEEE Eng Med Biol (0.14), IEEE Robot Autom Mag (0.13), Arch Phys Med Rehab (0.11), and IEEE T Neur Sys Reh (0.10). IEEE T Neur Sys Reh is a publication of the IEEE Engineering in Medicine and Biology Society, with broad subjective terms, including biomedical engineering and physical and rehabilitation medicine. J Rehabil Res Dev particularly focuses on physical and rehabilitation medicine, self-help devices, and rehabilitation research and development. These two journals, both of which have a high co-citation count and high centrality, were identified as influential journals and play an important role in EPW HMI methodology research. They tend to play an important role in research by providing a substantial reference to the scholars focusing on EPW HMI methodology.

### 4.2. Mapping and Analysis by Country and Institution

It is easy to find leading productive countries and active research teams involved in EPW HMI methodology research worldwide using a country and institution analysis. According to our database from Table 3, the United States with the biggest circle is the most productive country, with 240 publications, followed by China (134), Japan (98), and the UK (70).

**Figure 4:**
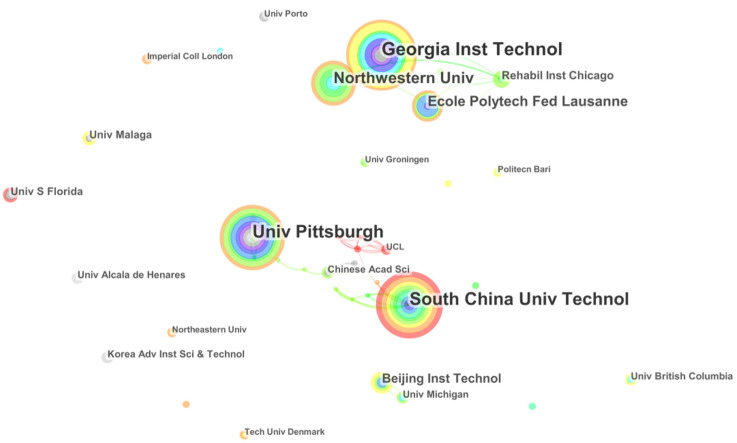
An institution collaboration map related to EPW HMI methodology research from 1998 to 2020.

The institution collaboration network consisted of 85 institutions and 49 collaboration links from 1998 to 2020 (Figure 4). The top 10 institutions that made the majority of contributions to the total output are listed in Table 4. A strong collaboration offers hints that two or more institutions are more likely to keep steady research investments to the specified fields. The Georgia Institute of Technology is the most contributive institute, with 29 publications, and cooperates with other institutions, namely Northwestern University and the Rehabilitation Institute of Chicago. The United States is the largest contributor to EPW HMI methodology research, with five institutions: the Georgia Institute of Technology, the University of Pittsburgh, Northwestern University, the Rehabilitation Institute of Chicago, and the University of South Florida, ranked 1, 3, 4, 7, and 8, respectively. China is another contributing country, with three institutions: the South China University of Technology, the Beijing Institute of Technology, and the Chinese Academy of Sciences. Corresponding to the result of the performance of countries and institution, it is showing that the USA is the largest contributor in the research of EPW HMI methodology with a leading position. Additionally, other countries, such as China, Japan, the UK, India, are very important complements to promote the developments.

These results may be as sociated with the introduction of policy and legislation, the establishment of disabled persons’ organizations and institutions, and the implementation of disabled assistance schemes and programs in these countries. Alternative funding schemes, such as governmental subsidies and low-interest-rate loan programs, would help the disabled obtain appropriate wheelchairs in the United States [26]. The Fourteenth Five-Year Plan of China proposes research topics in the field of assistive devices for the disabled, including foldable, portable electric wheelchairs [27]. A key piece of legislation in the UK is the Disability Discrimination Act (DDA) of 1995, which is loosely associated with building regulations to widen doorways for wheelchair access [28].

### 4.3. Mapping and Analysis by Author and Citied Author

**Figure 5:**
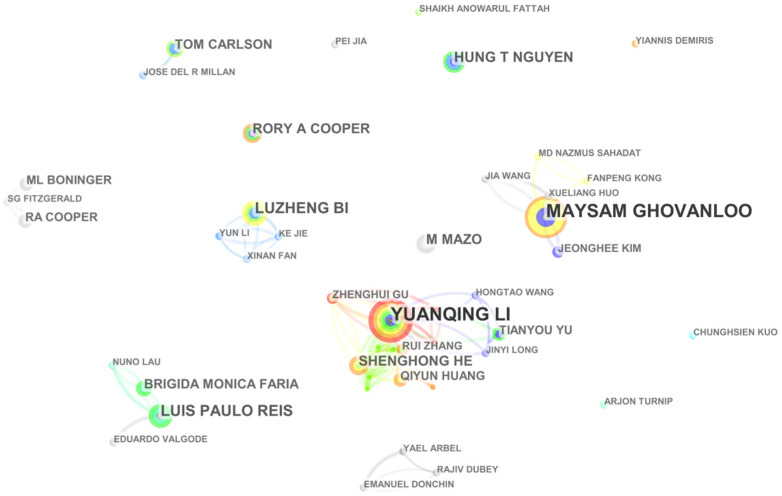
A co-author map related to EPW HMI methodology research from 1998 to 2020.

Knowledge mapping provides information related to potential collaborators and productive and influential researchers. The co-author map contributing to EPW HMI methodology research is presented in Figure 5, with 136 authors and 216 collaboration links. The size of the circle represents the number of publications, and circles of the same color represent authors of the same cluster. According to Figure 5, many researchers tend to cooperate with a relatively stable group of collaborators to generate several major clusters. Several collaborations among Y. Q. Li, J. Y. Long, H. T. Wang, and T. Y. Yu; Y. Q. Li, Q. Y. Huang, and S. H. He; M. Ghovanloo, M. N. Sahadat, F. P. Kong; and M. Ghovanloo, X. L. Huo, and J. Wang were observed from the chains among them in Figure 5.

The top 10 most productive authors are listed in Table 5. Y. Q. Li, evidently, is the most productive researcher in the field of EPW HMI methodology, based at the South China University of Technology in China, focusing on brain-controlled wheelchairs. He proposed a hybrid BCI system combining the mu rhythm and the P300 potential based on motor images for simulated wheelchair direction control. A hybrid BCI system combining P300 and the steady-state visual potential (SSVEP) was developed to enhance real-time asynchronous wheelchair control. In addition, a novel HMI based on eye movement was proposed for wheelchair control: the graphical user interface (GUI) includes 13 flashing buttons, and the user inputs control commands by blinking in sync with button flashes [29,30,31]. M. Ghovanloo is another productive researcher, with 15 publications in our database. He has worked on tongue-drive-assistive technology and implantable medical devices for over 10 years. He was an editorial board member for the IEEE Transaction on Biomedical Circuits and Systems and IEEE Transactions on Biomedical Engineering. Collaborating with J. Kim et al., he found that the TDS has faster speed and higher accuracy than traditional assistive technologies such as SnP [7,8,32,33]. Cooperating with X. L. Huo et al., he developed a customized interface circuit with an external TDS prototype for EPW driving [34]. Collaborating with M. N. Sahadato et al., he proposed a support vector machine based on linear accounting for high TDS computational efficiency and independent device control [35].

**Figure 6:**
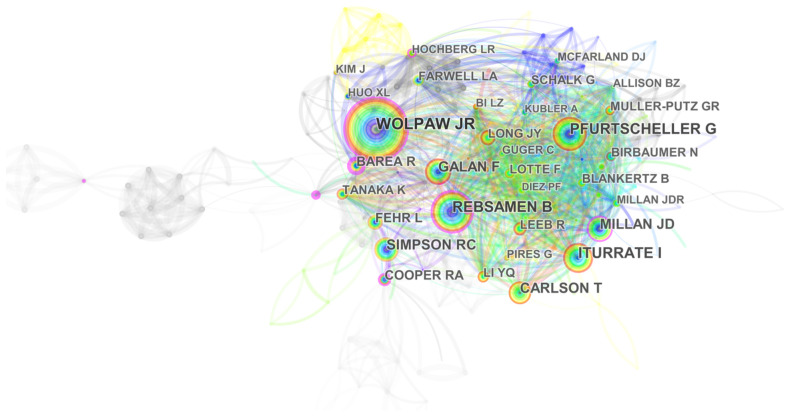
Author co-citation map related to EPW HMI methodology research from 1998 to 2020.

The author co-citation map related to EPW HMI methodology research from 1998 to 2020 is shown in Figure 6, with 238 nodes and 1447 co-citation links. Table 6 lists the top five authors with co-citation counts greater than 111 or a centrality greater than 0.13. J. R. Wolpaw, at the Laboratory for Neural Injury and Repair, New York State Department of Health, with the biggest circle in Figure 6, is the most influential researcher due to his high co-citation counts, focusing on the development of BCI for people with mobility impairment [36,37,38]. In terms of centrality, the top five authors are R. Barea, M. Mazo, G. Bourhis, R. A. Cooper, and B. Rebsamen. R. Barea proposed a modular eye control method based on EOG for meeting the mobile and communication needs of specific users. He also developed an EOG eye model based on wavelet transform and neural networks for HMI, which generated acceptable manipulative drifts for long term usage [3,39,40]. These analyses of the performance of authors show the most active and fruitful authors in the field in quantity of publications.

### 4.4. Mapping and Analysis by Reference

**Figure 7:**
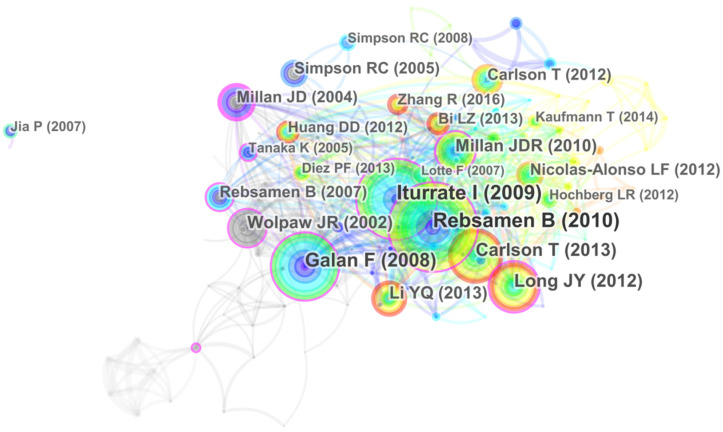
Reference co-citation map related to EPW HMI methodology research from 1998 to 2020.

Reference analysis examines the network of co-cited references to obtain key articles contributing to the field of EPW HMI methodology. The most cited articles are usually considered landmarks due to their ground-breaking contributions [41]. Figure 7 shows the reference co-citation map consisting of 152 references cited and 612 co-citation links from 1998 to 2020. Table 7 outlines the top 10 most cited references with a co-citation count of over 30. The author co-citation map indicates information, which is different from the reference co-citation map; the former emphasizes the key researcher of the field, while the latter highlights the masterpiece of work that impacts the research field.

In the network of Figure 7, nodes represent the cited situation of documents, and links represent the co-citation relations between one node and another. The larger the node, the higher frequency of citation of the document, indicating that the document is of great importance in EPW HMI methodology. “A Brain Controlled Wheelchair to Navigate in Familiar Environments” co-authored by B. Rebsamen et al. with the biggest circle in Figure 7 is the most cited article in our dataset, with 87 citations. Their work proposed a brain-controlled wheelchair (BCW) with a slow but reliable P300-based BCI for destination selection and a faster BCI for stopping. Their BCW enabled users to move to various locations in a familiar environment in less time and with significantly less control effort, emphasizing the importance of safety and efficiency in EPW HMI [42]. The second-most frequently co-cited article is by I. Iturrate et al., describing a non-invasive brain-actuated wheelchair with a P300 neurophysiological protocol and automated navigation, giving users the flexibility to use the device in unknown and evolving scenarios. Their overall results showed the great adaptation, high robustness, and low variability of the system [9]. In [43,44], researchers used an asynchronous non-invasive BCI to control a wheelchair or mobile robot. There are two types of protocols, synchronous and asynchronous, used for EEG-based wheelchair control. The synchronous protocol fixes a direction before reaching the destination, while the asynchronous protocol allows the user to interact with the wheelchair spontaneously rather than having to wait for external cues, providing the possibility of continuous control. T. Carlson et al. proposed a shared control architecture that combines the intelligence and desires of users with the accuracy of electric wheelchairs, emphasizing the importance of human factors in wheelchair evaluation. They used an asynchronous motor imagery-based BCI protocol for spontaneous wheelchair control and concluded that although the training procedure for motor-imagery-based BCIs might take longer than that for stimulus-driven P300 systems, it is ultimately meaningful and rewarding [45,46]. In [10], researchers introduced a hybrid BCI combining the motor-imagery-based mu rhythm and the P300 potential to control the direction and speed of the wheelchair, while, in [30], researchers proposed a hybrid BCI system combining the P300 potential and the SSVEP to improve the performance of asynchronous wheelchair control. Their results demonstrated that, with the use of two or more brain patterns, hybrid BCIs can reach their destinations more effectively than conventional BCI systems. In [17], researchers focused on the application of BCIs and identified four application areas where the disabled can greatly benefit from advancements in BCI technology, namely communication and control, motor substitution, entertainment, and motor recovery. They reviewed the current state of BCI applications and proposed their expectations for development in key areas such as the design of hybrid BCI architectures, the conception of adaptation algorithms, the exploitation of mental states, the incorporation of human-computer interaction principles, and the development of novel BCI technology and EEG devices. In [47], researchers reviewed present-day BCIs and pointed out key issues for the future of BCI-based communication and control, including the extent to which this control depends on normal brain function, identification of the most fitted feature-extraction approach for translating these features into device control commands, and the adoption of precise and objective procedures for evaluating BCI performance.

It can be seen from these analyses that the landmark studies on EPW HMI methodology research mostly focus on BCWs. These studies conform the critical role and widespread application of BCI in EPW HMI, indicate the main neurophysiological protocols used in BCI-based HMI, and reflect the continuous improvement of BCI-based EPW HMI performance. Two studies introducing the shared control architecture demonstrate that man-machine collaborative control is an adoptive form of cooperation between humans and machines in EPW HMI because it fuses multiple information sources, decides on appropriate maneuvers for execution, takes advantage of the respective benefits of humans and machines, and reduces the workload of human operators. In addition, safety, efficiency, accuracy, and user workload were important metrics for EPW HMI evaluation in these studies, showing that traditional machine performance and human factors are both considerations in determining the success of EPW HMI design.

**Table 7:** Top 10 most cited references in EPW HMI methodology research.

Ranking	Author	Year	Reference	Citation Count
1	B. Rebsamen	2010	A Brain Controlled Wheelchair to Navigate in Familiar Environments [42]	87
2	I. Iturrate	2009	A Noninvasive Brain-Actuated Wheelchair Based on a P300 Neurophysiological Protocol and Automated Navigation [9]	79
3	F. Galan	2008	A Brain-Actuated Wheelchair: Asynchronous and Non-invasive Brain-Computer Interfaces for Continuous Control of Robots [43]	66
4	T. Carlson	2013	Brain-Controlled Wheelchairs: A Robotic Architecture [45]	54
5	J. Y. Long	2012	A Hybrid Brain Computer Interface to Control the Direction and Speed of a Simulated or Real Wheelchair [10]	52
6	J. D. R. Millan	2010	Combining Brain-Computer Interfaces and Assistive Technologies: State-of-the-Art and Challenges [17]	37
7	Y. Q. Li	2013	A Hybrid BCI System Combining P300 and SSVEP and Its Application to Wheelchair Control [30]	37
8	J. R. Wolpaw	2002	Brain–Computer Interfaces for Communication and Control [47]	36
9	T. Carlson	2012	Collaborative Control for a Robotic Wheelchair: Evaluation of Performance, Attention, and Workload [46]	31
10	J. D. Millan	2004	Noninvasive Brain-Actuated Control of a Mobile Robot by Human EEG [44]	30

### 4.5. Mapping and Analysis by Keyword

Keyword analysis is used to gain more insight into the substance of a field and can identify current research hotspots and future directions [41]. CiteSpace can detect keywords with the highest frequency and align them based on their appearance time. These functions can be used to depict the knowledge structure of focus and potential future trends visually. To reduce noise, we first combined words with the same meaning, such as brain-computer interface; electroencephalogram (EEG), and electroencephalogram; people and individual; disabled person and disabled people.

**Figure 8:**
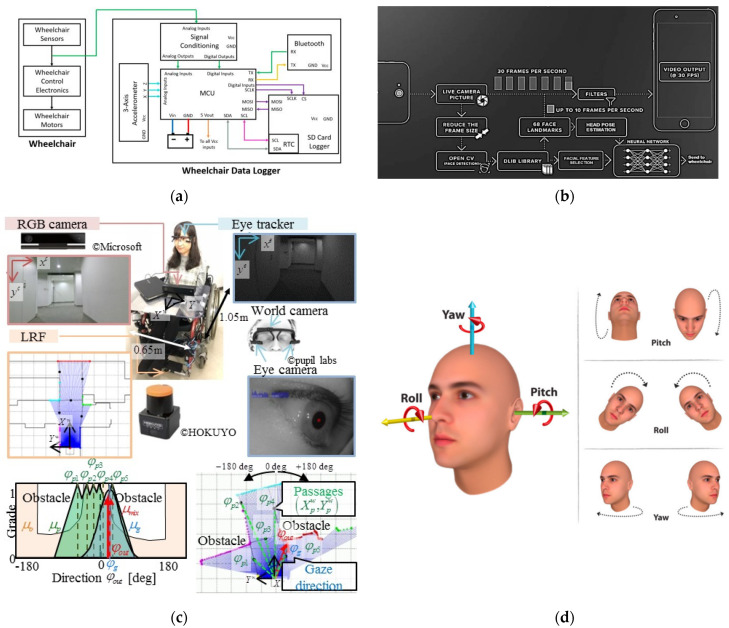
Examples of the remarkable technological advances in EPW HMI methodology in recent years. (**a**) A wheelchair data logger: proposed to capture data from human-wheelchair interaction for the head-foot steering system [54] (Reprinted with permission from ref. [54]. Copyright 2021 MDPI); (**b**) A facial expression-controlled wheelchair: based on a combination of neural networks (NN) and specific image preprocessing steps, providing an efficient hands-free option, allowing the user to drive the wheelchair using its facial expressions which can be flexibly updated [66] (Reprinted with permission from ref. [66]. Copyright 2021 Elsevier); (**c**) An Eye-controlled wheelchair system: based on gaze detection and environment recognition, allowing the passenger to move in the unknown environment by gazing towards the direction he or she wants to [61] (Reprinted with permission from ref. [61]. Copyright 2021 MDPI); (**d**) A Robotic Wheelchair operated using the head gesture: detecting the movement of the head, transmitting and processing the signal for mobility assistance, designed in a cost-effective way but ensures safety, flexibility, and mobility [67] (Reprinted with permission from ref. [67]. Copyright 2021 Elsevier).

Table 8 lists the top 20 keywords in terms of their frequency in EPW HMI methodology research, while Table 9 classifies them into four dimensions—technology, information, people, and society—based on the I-model proposed in [48]. Both Table 8 and Table 9 show more hotspots in general, focusing on the dimensions of technology and information rather than people and society. Among these keywords, BCI (271) is the most used word, reflecting the importance and popularity of BCI technology in EPW HMI methodology research, consistent with the previous observations of landmarks. Besides BCI, we also found that, within the technology and information dimensions, researchers focus more on the application of communication protocols, assistive technology, and classification algorithms to improve the performance of EPW HMI. In addition, researchers have used technologies originally developed for mobile robots to create smart wheelchairs that reduce the physical, perceptual, and cognitive skills necessary to operate a power wheelchair for individuals with severe dysfunction disorders such as amyotrophic lateral sclerosis (ALS), spinal cord injury (SCI), and muscle dystrophy (MS) [13,49]. Different kinds of input methods, such as joysticks [2,50], voice commands [51,52], the sip-and-puff interface [6], BCI [9,10,17,43], the tongue drive system (TDS) [7,8,32,33,34,35], the head gesture based interface (HGI) [53,54], the eye-controlled interface [3,39,55,56,57,58,59,60,61], the EMG-based interface [62,63], and the multimodal interface [64,65], have been used in EPW HMI to accommodate the disabled. Some examples of the remarkable technological advances in EPW HMI methodology in recent years are shown in detail in Figure 8.

**Figure 9:**
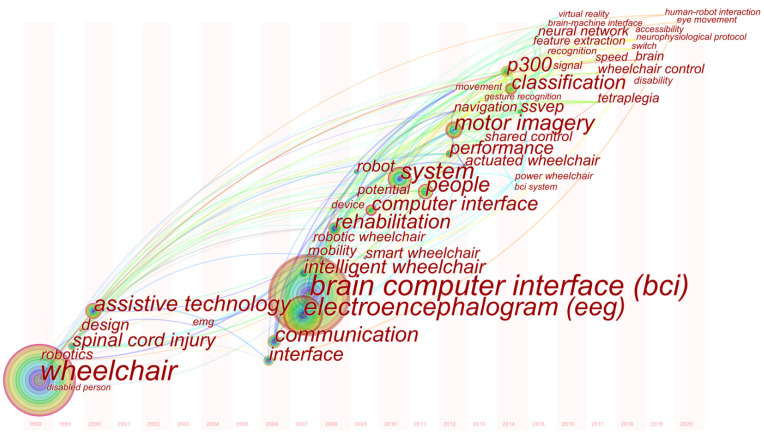
Keyword co-occurrence time zone map for EPW HMI methodology research.

To determine the evolution of the research focus, a time zone view of the keywords is illustrated in Figure 9, and this visualization arranges the keywords according to the time of their first appearance. EOG and EMG were used as input interfaces for EPW HMI before 2007 [39,68]. The extensive appearance of intelligent wheelchairs, BCI, and EEG in 2007, consistent with the first sharp increase in EPW HMI methodology research publications stated in Section 4, indicates that research on brain-controlled wheelchairs has flourished since 2007 due to the advancement of BCI technology and EEG application. Motor imagery, P300, and SSVEP appeared in 2012, 2014, and the same year 2014, respectively, indicating that research has focused on further exploration of BCI-based HMI to realize better humanized control (continuous control and prevention on fatigue driving) of wheelchairs [9,10,30,45,69,70,71]. The appearance of signals, recognition, feature extraction, classification, and neural networks focusing on information processing and shared control focusing on control strategies proves that not only machine performance (efficiency and accuracy) but also human factors (comfort and independence) are increasingly considered in EPW HMI research [72,73,74,75,76,77,78,79]. The relatively large nodes of the time zone map in recent years, such as speed, tetraplegia, brain, switch, eye movement, and human-robot interaction, demonstrate that speed control (high speed, low speed) and state (moving forward, stopped) or control mode switching [80,81,82,83], the application of computer technology (neural network, deep learning) [84], the development of novel EOG-based HMI [85], and the accessibility of people with tetraplegia [86], have become hot topics in recent years.

In summary, according to the evolution of the research focus, it can be found that more research has focused on humanized control (smooth continuous control and prevention on fatigue driving) and the addition of human intention in a control framework and strategy. Therefore, we believe that an intelligentized and humanized EPW HMI system based on human-machine collaborative control is emerging to be a hot trend in years to come. To clarify, “intelligentize” denotes a gradual integration of computer and robotics technology, to optimize the autonomous property in control and interaction continuously. In this work, the term “humanization” or “humanizing” refers to a design processing that respects and considers human patterns such as behavior, physiological character, and psychological factors. It refers to a hybrid evolution of design and engineering process, from functional and technological aspects to human-centered methodology, including but not limited to usability, feasibility and user experience designing. Specifically, the trend of humanization is observed for EPW HMI, using essential aspects of Nielsen usability principles, such as “User control and freedom”, “Consistency and standards”, “Recognition rather than recall”, have been valued in the design process. Human-machine collaboration means that the human-machine active-passive hybrid mode based on a shared control strategy is to replace the conventional human-dominated control mode, which empowers users with extra degrees of freedom. This mode not only plays a supporting role but also preserves part of the users’ independence. In addition, technological advances permit data acquisition by means of wireless, mobile, wearable, and low-cost devices, providing a possibility of a wider range of daily-life applications of EPW HMI methodology. At the same time, new aspects not considered before have become challenges that we must face to translate these interaction methods from proof-of-concept prototypes to reliable applications. In terms of the application of BCI, the roadblocks faced on the translation include safety and biocompatibility of invasive approaches, as well as wearability and ergonomics of non-invasive recording techniques. In the latter case, the main reason preventing EEG-based BCIs from being widely used is arguably their poor usability, which is notably due to their low robustness and reliability, as well as their often long calibration and training times, although, BCIs based on P300, SSVEP generally require shorter training periods [16,87]. Fortunately, some researchers have been making efforts to accelerate the application of BCI. Minguillon J et.al presented a critical review of EEG artifact removal approaches and gave some directions and guidelines (use multiple-step procedures, define and characterize most of artifacts evoked in daily-life EEG-BCI) for upcoming research [88]. Chavarriaga R et al. summarized several methodological aspects (integrate the user’s needs and preferences into the design of the BCI solution; overcome pitfalls at the user training level; be cautious on the application and evaluation of digital signal processing and pattern recognition methods; and embrace the interdisciplinary nature of BCI) that need to be taken into account in order to deploy BCI systems to intended end-users successfully [89]. The EPW HMI methodology still has a long way to go from the laboratories to life scenarios. How to provide more efficient, smart, and considerate services to end-users, how to evaluate the applicability and benefits of the EPW HMI methodology for the disabled, and how much contribution it brings to society will become interesting questions in the future.

## 5. Conclusions

This paper applied CiteSpace as a bibliometric analytical tool to conduct an analysis of EPW HMI methodology research. Based on bibliometric analysis, this article analyzed 1154 publications related to EPW HMI methodology from 1998 to 2020, retrieved from the WOS database, and identified the research status of and future potential research trends in this field.

A number of insights can be drawn from the results. First, research on the EPW HMI methodology has gained a lot of ground since 1998. The research and development of this field is an interdisciplinary problem, requiring multidisciplinary collaboration. Second, research on the EPW HMI methodology is dominated by the United States, China, Japan, and the UK because of the introduction of policies and legislation, the establishment of organizations and institutions, and the implementation of assistance programs. Institutions and researchers from these countries have made great contributions to this research field. Third, according to the bibliometric study, the most studied EPW HMI technology is BCI. Being different from other primary HMI methods, BCI is purely in the laboratory stage instead of being available on the real market. Other HMI methods, such as voice driving, eye tracking, SnP, TDS, and EMG control, are also used in EPW HMI to accommodate the users. Although BCI gains most attention in current research, it has relatively few successful cases in practical applications. Finally, in general, hotspots focus on the dimensions of technology and information rather than people and society. Increasingly hot topics include the enhancement of interaction system performance and the integration of human factors over time. Based on the evolution of the research foci, we believe that research around intellectualization and humanization are emerging among the top trends in the context of EPW HMI research. In summary, based on the main findings discussed above, we believe that researchers may find new insights into the combination of intelligence and the humanization of EPW HMI systems based on the human-machine collaboration, making more theoretical, methodological, and practical contributions.

It is worth noting that HMIs without considering application and practice scenarios are meaningless. For example, at the practical level, the affordable solution with best efforts on usability is a primary goal, e.g., joysticks, audio, and haptics; at the laboratory level, the fancy HMI that embraces the latest progress from interdisciplinary collaboration towards achieving a feat that balances the human-machine cooperative perception, information conveying capability and efficiency, as well as user experience, without much consideration of commercialization, e.g., eye-sight tracing, extracorporeal BCI, EEG; at the prospective level, there would be numerous attempts not only to borrow strength from relevant research fields but also to conduct fundamental research towards forward-thinking technology and methodology to enhance the HMI in EPW, e.g., implantable BCI, wireless EEG.

Previous studies have investigated how to enhance the performance of EPW HMI in different ways from the dimensions of technology and information. From the dimensions of people and society, how to evaluate the applicability and benefits of the EPW HMI methodology for the disabled and how much contribution it brings to society will become an interesting question in the future.

Compared to existing research in the field, we combined network maps and information tables to conduct an objective, systematic quantitative analysis; summarized previous research; and provided possible research trends for readers. Our study gives insight into EPW HMI methodology research, provides valuable information for upcoming researchers, and hopefully promotes relevant assistive research for the elderly and the disabled. The contributions of this work include: (1) To the best of our knowledge, our work is the first up-to-date systematical review work on relevant HMI for the EPW which is essential for the EPW human-machine loop and socio-technical meaning; (2) This work interprets the EPW HMI objective quantitative bibliometric analysis, which differs from the conventional qualitative review. It does not only summarize merits of state-of-art works but also predicts possible research trends for readers who are new to the field; (3) This work focuses not only on the aspects of engineering and intelligence, but also includes relevant works from the field of socio-technical system design and interaction design. Compared with review works from a purely engineering viewpoint, our work is solid regarding interdisciplinary research and cooperation.

However, there are still some inherent limitations of this study, and researchers could focus on the following areas in the future: (1) Researchers tending to cite their own publications may affect co-citation analysis results. Self-citation should be accounted for and eliminated in future research. (2) Future research may consider analyzing more comprehensive publications (e.g., English and non-English literature) related to the EPW HMI methodology to arrive at more robust conclusions.

## Figures and Tables

**Table 1:** Document types for publications referencing electric power wheelchair (EPW) human-machine interaction (HMI) methodology.

Ranking	Type	Count (%)
1	Proceeding paper	662 (57.37)
2	Article	451 (39.08)
3	Review	38(3.29)
4	Letter	2 (0.17)
5	Meeting abstract	1 (0.09)

**Table 2:** Top five co-cited journals related to EPW HMI methodology research in terms of co-citation counts and centrality.

Ranking	Co-Citation Count	Co-Cited Journal	Impact Factor	Centrality	Co-Cited Journal	Impact Factor
1	469	IEEE T Neur Sys Reh	4.003	0.19	J Rehabil Res Dev	2.235
2	365	IEEE T Bio-Med Eng	4.820	0.14	IEEE Eng Med Biol	1.526
3	272	Clin Neurophysiol	3.657	0.13	IEEE Robot Autom Mag	4.615
4	263	J Rehabil Res Dev	2.235	0.11	Arch Phys Med Rehab	3.690
5	248	J Neural Eng	4.473	0.10	IEEE T Neur Sys Reh	4.003

Impact factor: average impact factor (5 years).

**Table 3:** Top 10 countries involved in EPW HMI methodology research.

Ranking	Country	Publications	Centrality
1	United States	240	0.01
2	China	134	0.01
3	Japan	98	0.07
4	UK	70	0.20
5	India	59	0.17
6	South Korea	41	0
7	Spain	36	0
8	France	34	0.27
9	Italy	31	0.11
10	Canada	29	0.02

**Table 4:** Top 10 institutions involved in EPW HMI methodology research.

Ranking	Institution	Country	Publications	Centrality
1	Georgia Institute of Technology	United States	29	0
2	South China University of Technology	China	27	0.05
3	University of Pittsburgh	United States	27	0.02
4	Northwestern University	United States	18	0
5	École Polytechnique Fédérale de Lausanne	Switzerland	13	0
6	Beijing Institute of Technology	China	9	0
7	Rehabilitation Institute of Chicago	United States	7	0
8	University of South Florida	United States	6	0
9	Universidad de Málaga	Spain	6	0
10	Chinese Academy of Sciences	China	5	0.04

**Table 5:** Top 10 most productive authors involved in EPW HMI methodology research.

Ranking	Publications	Author	Ranking	Publications	Author
1	16	Yuanqing Li	6	7	Rory A. Cooper
2	15	Maysam Ghovanloo	7	7	Shenghong He
3	9	Luis Paulo Reis	8	7	M. Mazo
4	9	Luzheng Bi	9	6	Tom Carlson
5	8	Hung T. Nguyen	10	6	Brigida Monica Faria

**Table 6:** Top 5 co-cited authors involved in EPW HMI methodology research in terms of co-citation count and centrality.

Ranking	Co-Citation Count	Cited Author	Centrality	Cited Author
1	220	J. R. Wolpaw	0.35	R. Barea
2	159	B. Rebsamen	0.29	M. Mazo
3	146	G. Pfurtscheller	0.27	G. Bourhis
4	132	I. Iturrate	0.18	R. A. Cooper
5	111	F. Galan	0.13	B. Rebsamen

**Table 8:** Top 20 keywords in terms of their frequency in EPW HMI methodology research.

Ranking	Keyword	Frequency	Dimension
1	BCI	271	Technology
2	Wheelchair	254	Technology
3	EEG	141	Information
4	System	85	All
5	Assistive technology	63	Technology
6	Motor imagery	62	technology
7	People	59	People
8	Communication	56	Information
9	Rehabilitation	47	Society
10	Computer interface	43	Technology
11	Classification	43	Information
12	P300	42	Information
13	Intelligent wheelchair	39	Technology
14	Interface	38	Technology
15	Spinal cord injury	36	People
16	Performance	32	Technology
17	SSVEP	21	Information
18	Robot	21	Technology
19	Design	20	Society
20	Actuated wheelchair	16	Technology

**Table 9:** Keywords of EPW HMI methodology research in the I-model.

Technology Aspect	Information Aspect	People Aspect	Society Aspect
BCI	EEG	System	System
Wheelchair	System	People	Rehabilitation
System	Communication	Spinal cord injury	Design
Intelligent wheelchair	P300		
Assistive technology	Classification		
Motor imagery	SSVEP		
Interface			
Actuated wheelchair			
Computer interface			
Performance			
Robot			

## Data Availability

Data sharing not applicable.

## References

[1] World Health Organization Assistive Technology. https://www.who.int/news-room/fact-sheets/detail/assistive-technology.

[2] Cooper R.A., Spaeth D.M., Jones D.K., Boninger M.L., Fitzgerald S.G., Guo S. (2002). Comparison of virtual and real electric powered wheelchair driving using a position sensing joystick and an isometric joystick. Med. Eng. Phys..

[3] Barea R., Boquete L., Mazo M., López E. (2002). Wheelchair Guidance Strategies Using EOG. J. Intell. Robot. Syst..

[4] Tsui C.S.L., Jia P., Gan J.Q., Hu H., Yuan K. EMG-based hands-free wheelchair control with EOG attention shift detection. Proceedings of the 2007 IEEE International Conference on Robotics and Biomimetics (ROBIO).

[5] Mónica Faria B., Vasconcelos S., Paulo Reis L., Lau N. (2013). Evaluation of distinct input methods of an intelligent wheel-chair in simulated and real environments: A performance and usability study. Assist. Technol..

[6] Grewal H.S., Matthews A., Tea R., Contractor V., George K. Sip-and-Puff Autonomous Wheelchair for Individuals with Severe Disabilities. Proceedings of the 9th IEEE Annual Ubiquitous Computing, Electronics & Mobile Communication Conference (UEMCON).

[7] Kim J., Park H., Bruce J., Sutton E., Rowles D., Pucci D., Holbrook J., Minocha J., Nardone B., West D. (2013). The Tongue Enables Computer and Wheelchair Control for People with Spinal Cord Injury. Sci. Transl. Med..

[8] Kim J., Park H., Bruce J., Rowles D., Holbrook J., Nardone B., West D.P., Laumann A.E., Roth E., Veledar E. (2014). Qualitative assessment of tongue drive system by people with high-level spinal cord injury. J. Rehabil. Res. Dev..

[9] Iturrate I., Antelis J.M., Kubler A., Minguez J. (2009). A Noninvasive Brain-Actuated Wheelchair Based on a P300 Neurophysiological Protocol and Automated Navigation. IEEE Trans. Robot..

[10] Long J., Li Y., Wang H., Yu T., Pan J., Li F. (2012). A Hybrid Brain Computer Interface to Control the Direction and Speed of a Simulated or Real Wheelchair. IEEE Trans. Neural Syst. Rehabil. Eng..

[11] Lebedev M. (2014). Brain-machine interfaces: An overview. Transl. Neurosci..

[12] Phinyomark A., Limsakul C., Phukpattaranont P. (2011). A Review of Control Methods for Electric Power Wheelchairs Based on Electromyography Signals with Special Emphasis on Pattern Recognition. IETE Tech. Rev..

[13] Simpson R.C. (2005). Smart wheelchairs: A literature review. J. Rehabil. Res. Dev..

[14] Leaman J., La H.M. (2017). A comprehensive review of smart wheelchairs: Past, present and future. IEEE Trans. Hum. Mach. Syst..

[15] Van Leeuwen T. (2006). The application of bibliometric analyses in the evaluation of social science research. Who benefits from it and why it is still feasible. Scientometrics.

[16] Guger C., Daban S., Sellers E., Holzner C., Krausz G., Carabalona R., Gramatica F., Edlinger G. (2009). How many people are able to control a P300-based brain–computer interface (BCI)?. Neurosci. Lett..

[17] Millán J.D.R., Rupp R., Mueller-Putz G., Murray-Smith R., Giugliemma C., Tangermann M., Mattia D. (2010). Combining brain–computer interfaces and assistive technologies: State-of-the-art and challenges. Front. Neurosci..

[18] Chen D., Liu Z., Luo Z., Webber M., Chen J. (2016). Bibliometric and visualized analysis of emergy research. Ecol. Eng..

[19] Ma R., Ho Y.-S. (2016). Comparison of environmental laws publications in Science Citation Index Expanded and Social Science In-dex: A bibliometric analysis. Scientometrics.

[20] Liang Y.-D., Li Y., Zhao J., Wang X.-Y., Zhu H.-Z., Chen X.-H. (2017). Study of acupuncture for low back pain in recent 20 years: A bibliometric analysis via CiteSpace. J. Pain Res..

[21] Gao X.-L., Sui Y., Liu S., Sun Y.-P. (2019). Knowledge domain and emerging trends in Alzheimer’s disease: A scientometric review based on CiteSpace analysis. Neural Regen. Res..

[22] Fahimnia B., Sarkis J., Davarzani H. (2015). Green supply chain management: A review and bibliometric analysis. Int. J. Prod. Econ..

[23] Chen C. (2004). Searching for intellectual turning points: Progressive knowledge domain visualization. Proc. Natl. Acad. Sci. USA.

[24] Chen C. The centrality of pivotal points in the evolution of scientific networks. Proceedings of the 10th International Con-ference on Intelligent User Interfaces.

[25] Chen C. (2006). CiteSpace II: Detecting and visualizing emerging trends and transient patterns in scientific literature. J. Am. Soc. Inf. Sci. Technol..

[26] Hunt P.C. (2005). Factors Associated with Wheelchair Use and the Impact on Quality of Life among Individuals with Spinal Cord Injury. Ph.D. Dissertation.

[27] The “Fourteenth Five-Year Plan” Key Research Project for the Disabled in the Early Stage and the 2019 China Disabled Persons’ Federation Research Project Announcement. http://www.cdpf.org.cn/ggtz/201908/t20190813_659708.shtml.

[28] Roulstone A., Prideaux S. (2009). Constructing reasonableness: Environmental access policy for disabled wheelchair users in four European Union countries. Alter.

[29] Long J.J., Li Y.Y., Wang H.H., Yu T.T., Pan J.J. Control of a simulated wheelchair based on a hybrid brain computer interface. Proceedings of the 2012 Annual International Conference of the IEEE Engineering in Medicine and Biology Society.

[30] Li Y., Pan J., Wang F., Yu Z. (2013). A Hybrid BCI System Combining P300 and SSVEP and Its Application to Wheelchair Control. IEEE Trans. Biomed. Eng..

[31] Huang Q., He S., Wang Q., Gu Z., Peng N., Li K., Zhang Y., Shao M., Li Y. (2018). An EOG-Based Human–Machine Interface for Wheelchair Control. IEEE Trans. Biomed. Eng..

[32] Kim J., Park H., Bruce J., Rowles D., Holbrook J., Nardone B., Ghovanloo M. (2015). Assessment of the tongue-drive system using a computer, a smartphone, and a powered-wheelchair by people with tetraplegia. IEEE Trans. Neural Syst. Rehabil. Eng..

[33] Kim J., Huo X., Minocha J., Holbrook J., Laumann A., Ghovanloo M. (2012). Evaluation of a Smartphone Platform as a Wireless Interface Between Tongue Drive System and Electric-Powered Wheelchairs. IEEE Trans. Biomed. Eng..

[34] Huo X., Wang J., Ghovanloo M. Wireless control of powered wheelchairs with tongue motion using tongue drive assistive technology. Proceedings of the 30th Annual International Conference of the IEEE Engineering in Medicine and Biology Society.

[35] Sahadat M.N., Sebkhi N., Anderson D., Ghovanloo M. (2018). Optimization of tongue gesture processing algorithm for standalone multimodal tongue drive system. IEEE Sens. J..

[36] Wolpaw J.R., Birbaumer N., Heetderks W.J., McFarland D.J., Peckham P.H., Schalk G., Vaughan T.M. (2000). Brain-computer interface technology: A review of the first international meeting. IEEE Trans. Rehabil. Eng..

[37] Wolpaw J.R., Mcfarland D.J. (2004). Control of a two-dimensional movement signal by a noninvasive brain-computer interface in humans. Proc. Natl. Acad. Sci. USA.

[38] Shahriari Y., Vaughan T.M., McCane L.M., Allison B.Z., Wolpaw J.R., Krusienski D.J. (2019). An exploration of BCI performance variations in people with amyo-trophic lateral sclerosis using longitudinal EEG data. J. Neural Eng..

[39] Barea R., Boquete L., Mazo M., Lopez E. (2002). System for assisted mobility using eye movements based on electrooculography. IEEE Trans. Neural Syst. Rehabil. Eng..

[40] Barea R., Boquete L., Ortega S., López E., Rodríguez-Ascariz J. (2012). EOG-based eye movements codification for human computer interaction. Expert Syst. Appl..

[41] Chen C., Hu Z., Liu S., Tseng H. (2012). Emerging trends in regenerative medicine: A scientometric analysis in CiteSpace. Expert Opin. Biol. Ther..

[42] Rebsamen B., Guan C., Zhang H., Wang C., Teo C., Ang M.H., Burdet E. (2010). A Brain Controlled Wheelchair to Navigate in Familiar Environments. IEEE Trans. Neural Syst. Rehabil. Eng..

[43] Galán F., Nuttin M., Lew E., Ferrez P.W., Vanacker G., Philips J., Millán J.D.R. (2008). A brain-actuated wheelchair: Asynchronous and non-invasive brain–computer interfaces for continuous control of robots. Clin. Neurophysiol..

[44] Millan J.R., Renkens F., Mourino J., Gerstner W. (2004). Noninvasive brain-actuated control of a mobile robot by human EEG. IEEE Trans. Biomed. Eng..

[45] Carlson T., Millan J.D.R. (2013). Brain-controlled wheelchairs: A robotic architecture. IEEE Robot. Autom. Mag..

[46] Carlson T., Demiris Y. (2012). Collaborative control for a robotic wheelchair: Evaluation of performance, attention and workload. IEEE Trans. Syst. Man Cybern. Part B.

[47] Wolpaw J.R., Birbaumer N., McFarland D.J., Pfurtscheller G., Vaughan T.M. (2002). Brain–computer interfaces for communication and control. Clin. Neurophysiol..

[48] Zhang P., Benjamin R.I. (2007). Understanding information related fields: A conceptual framework. J. Am. Soc. Inf. Sci. Technol..

[49] Simpson R., Lopresti E., Hayashi S., Nourbakhsh I., Miller D. (2004). The smart wheelchair component system. J. Rehabil. Res. Dev..

[50] Cooper R.A., Jones D.K., Fitzgerald S., Boninger M.L., Albright S.J. (2000). Analysis of position and isometric joysticks for powered wheelchair driving. IEEE Trans. Biomed. Eng..

[51] Peixoto N., Nik H.G., Charkhkar H. (2013). Voice controlled wheelchairs: Fine control by humming. Comput. Methods Programs Biomed..

[52] Pires G., Nunes U. (2002). A wheelchair steered through voice commands and assisted by a reactive fuzzy-logic controller. J. Intell. Robot. Syst..

[53] Jia P., Hu H.H., Lu T., Yuan K. (2007). Head gesture recognition for hands-free control of an intelligent wheelchair. Ind. Robot. Int. J..

[54] Gakopoulos S., Nica I.G., Bekteshi S., Aerts J.-M., Monbaliu E., Hallez H. (2019). Development of a Data Logger for Capturing Human-Machine Interaction in Wheelchair Head-Foot Steering Sensor System in Dyskinetic Cerebral Palsy. Sensors.

[55] Matsumotot Y., Ino T., Ogsawara T. Development of intelligent wheelchair system with face and gaze based inter-face. Proceedings of the 10th IEEE International Workshop on Robot and Human Interactive Communication. ROMAN 2001.

[56] Barea R., Boquete L., Bergasa L.M., López E., Mazo M. (2003). Electro-oculographic guidance of a wheelchair using eye movements codification. Int. J. Robot. Res..

[57] Lin C.S., Ho C.W., Chen W.C., Chiu C.C., Yeh M.S. (2006). Powered wheelchair controlled by eye-tracking system. Opt. Appl..

[58] Eid M.A., Giakoumidis N., el Saddik A. (2016). A novel eye-gaze-controlled wheelchair system for navigating unknown environments: Case study with a person with ALS. IEEE Access.

[59] Ishida S., Takimoto M., Kambayashi Y. AR Based User Interface for Driving Electric Wheelchairs. Proceedings of the International Conference on Universal Access in Human-Computer Interaction.

[60] Ishizuka A., Yorozu A., Takahashi M. Motion control of a powered wheelchair using eye gaze in unknown environments. Proceedings of the 11th Asian Control Conference (ASCC).

[61] Ishizuka A., Yorozu A., Takahashi M. (2018). Driving control of a powered wheelchair considering uncertainty of gaze in-put in an unknown environment. Appl. Sci..

[62] Moon I., Lee M., Chu J., Mun M. Wearable EMG-based HCI for electric-powered wheelchair users with motor disabilities. Proceedings of the 2005 IEEE International Conference on Robotics and Automation.

[63] Wei L., Hu H., Zhang Y. (2011). Fusing EMG and visual data for hands-free control of an intelligent wheelchair. Int. J. Hum. Robot..

[64] Moon I., Lee M., Ryu J., Mun M. Intelligent robotic wheelchair with EMG-, gesture- and voice-based interfaces. Proceedings of the 2003 IEEE/RSJ International Conference on Intelligent Robots and Systems (IROS 2003).

[65] Reis L.P., Braga R.A., Sousa M., Moreira A.P. (2009). IntellWheels MMI: A flexible interface for an intelligent wheelchair. Robot Soccer World Cup.

[66] Rabhi Y., Mrabet M., Fnaiech F. (2018). A facial expression controlled wheelchair for people with disabilities. Comput. Methods Programs Biomed..

[67] Machangpa J.W., Chingtham T.S. (2018). Head gesture controlled wheelchair for quadriplegic patients. Procedia Comput. Sci..

[68] Han J.S., Bien Z.Z., Kim D.J., Lee H.E., Kim J.S. Human-machine interface for wheelchair control with EMG and its evaluation. Proceedings of the 25th Annual International Conference of the IEEE Engineering in Medicine and Biology Society.

[69] Diez P.F., Müllerc S.M.T., Mut V.A., Laciar E., Avila E., Bastos-Filho T.F., Sarcinelli-Filho M. (2013). Commanding a robotic wheelchair with a high-frequency steady-state visual evoked potential based brain–computer interface. Med. Eng. Phys..

[70] Cao L., Li J., Ji H., Jiang C. (2014). A hybrid brain computer interface system based on the neurophysiological protocol and brain-actuated switch for wheelchair control. J. Neurosci. Methods.

[71] Zhang R., Li Y., Yan Y., Zhang H., Wu S., Yu T., Gu Z. (2015). Control of a Wheelchair in an Indoor Environment Based on a Brain–Computer Interface and Automated Navigation. IEEE Trans. Neural Syst. Rehabil. Eng..

[72] Pushp S., Saikia A., Khan A., Hazarika S.M. (2018). A cognitively enhanced collaborative control architecture for an intelligent wheelchair: Formalization, implementation and evaluation. Cogn. Syst. Res..

[73] Saleh A.I., Shehata S.A., Labeeb L.M. (2019). A fuzzy-based classification strategy (FBCS) based on brain–computer interface. Soft Comput..

[74] Belwafi K., Gannouni S., Aboalsamh H., Mathkour H., Belghith A. (2019). A dynamic and self-adaptive classification algorithm for motor imagery EEG signals. J. Neurosci. Methods.

[75] Lotte F., Congedo M., Lécuyer A., Lamarche F., Arnaldi B. (2007). A review of classification algorithms for EEG-based brain–computer interfaces. J. Neural Eng..

[76] Nicolas-Alonso L.F., Gomez-Gil J. (2012). Brain computer interfaces: A review. Sensors.

[77] Lopes A.C., Pires G., Nunes U. (2013). Assisted navigation for a brain-actuated intelligent wheelchair. Robot. Auton. Syst..

[78] Millán J.D.R., Galán F., Vanhooydonck D., Lew E., Philips J., Nuttin M. Asynchronous non-invasive brain-actuated control of an intelligent wheelchair. Proceedings of the 2009 Annual International Conference of the IEEE Engineering in Medicine and Biology Society.

[79] Bi L., Fan X.-A., Liu Y. (2013). EEG-Based Brain-Controlled Mobile Robots: A Survey. IEEE Trans. Hum. Mach. Syst..

[80] Yu Y., Zhou Z., Liu Y., Jiang J., Yin E., Zhang N., Wang Z., Liu Y., Wu X., Hu D. (2017). Self-Paced Operation of a Wheelchair Based on a Hybrid Brain-Computer Interface Combining Motor Imagery and P300 Potential. IEEE Trans. Neural Syst. Rehabil. Eng..

[81] Bi L., Lu Y., Fan X., Lian J., Liu Y. (2016). Queuing Network Modeling of Driver EEG Signals-Based Steering Control. IEEE Trans. Neural Syst. Rehabil. Eng..

[82] Li Y., He S., Huang Q., Gu Z., Yu Z.L. (2018). A EOG-based switch and its application for “start/stop” control of a wheelchair. Neurocomputing.

[83] Yu Y., Liu Y., Jiang J., Yin E., Zhou Z., Hu D. (2018). An Asynchronous Control Paradigm Based on Sequential Motor Imagery and Its Application in Wheelchair Navigation. IEEE Trans. Neural Syst. Rehabil. Eng..

[84] Zhou X., Wang F., Wang J., Wang Y., Yan J., Zhou G. (2019). Deep Learning Based Gesture Recognition and Its Application in Interactive Control of Intelligent Wheelchair. Petri Nets and Other Models of Concurrency XV.

[85] Choudhari A.M., Porwal P., Jonnalagedda V., Mériaudeau F. (2019). An Electrooculography based Human Machine Interface for wheelchair control. Biocybern. Biomed. Eng..

[86] Sahadat N., Sebkhi N., Ghovanloo M. Simultaneous multimodal access to wheelchair and computer for people with tetraple-gia. Proceedings of the 20th ACM International Conference on Multimodal Interaction.

[87] Allison B., Luth T., Valbuena D., Teymourian A., Volosyak I., Graser A. (2010). BCI Demographics: How Many (and What Kinds of) People Can Use an SSVEP BCI?. IEEE Trans. Neural Syst. Rehabil. Eng..

[88] Minguillon J., Lopez-Gordo M.A., Pelayo F. (2017). Trends in EEG-BCI for daily-life: Requirements for artifact removal. Biomed. Signal Process. Control..

[89] Chavarriaga R., Fried-Oken M., Kleih S., Lotte F., Scherer R. (2017). Heading for new shores! Overcoming pitfalls in BCI design. Brain Comput. Interfaces.

